# Efficient Myogenic/Adipogenic Transdifferentiation of Bovine Fibroblasts in a 3D Bioprinting System for Steak‐Type Cultured Meat Production

**DOI:** 10.1002/advs.202202877

**Published:** 2022-10-03

**Authors:** Dayi Jeong, Jeong Wook Seo, Hong‐Gu Lee, Woo Kyung Jung, Yong Ho Park, Hojae Bae

**Affiliations:** ^1^ Department of Stem Cell and Regenerative Biotechnology KU Convergence Science and Technology Institute Konkuk University Seoul 05029 Republic of Korea; ^2^ Department of Animal Science and Technology Sanghuh College of Life Sciences Konkuk University Seoul 05029 Republic of Korea; ^3^ NoAH Biotech Co., Ltd. Suwon‐si Gyeonggi‐do 16614 Republic of Korea; ^4^ Department of Microbiology College of Veterinary Medicine and Research Institute for Veterinary Science Seoul National University 1 Gwanak‐ro, Gwanak‐gu Seoul 08826 Republic of Korea; ^5^ Institute of Advanced Regenerative Science Konkuk University 120 Neungdong‐ro, Gwangjin‐gu Seoul 05029 Republic of Korea

**Keywords:** 3D bioprinting, biofabrication, bioinks, cultured meat

## Abstract

The interest in cultured meat is increasing because of the problems with conventional livestock industry. Recently, many studies related to cultured meat have been conducted, but producing large‐sized cultured meat remains a challenge. It is aimed to introduce 3D bioprinting for producing large cell aggregates for cultured meat production. A hydrogel scaffold is produced at the centimeter scale using a bioink consisting of photocrosslinkable materials for digital light processing‐based (DLP) printing, which has high printing accuracy and can produce geometrically complex structures. The light exposure time for hydrogel photopolymerization by DLP bioprinting is optimized based on photorheometry and cell viability assays. Naturally immortalized bovine embryonic fibroblast cells transformed with *MyoD* and *PPARγ2* instead of primary cells are used as the latter have difficulties in maintaining stemness and are associated with animal ethics issues. The cells are mixed into the hydrogel for printing. Myogenesis and adipogenesis are induced simply by changing the medium after printing. Scaffolds are obtained successfully with living cells and large microchannels. The cooked cultured meat maintains its size and shape upon cutting. The overall dimensions are 3.43 cm × 5.53 cm × 0.96 cm. This study provides proof‐of‐concept for producing 3D cultured meat using bioinks.

## Graphical abstract

3D‐bioprinted steak‐type cultured meat production process using digital light processing‐based printing is suitable for solving the scalability challenges. The method allows not only using a simple mixture of muscle and fat cells but also controlling the muscle‐to‐fat ratio.

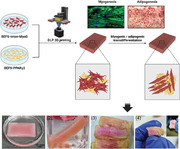

## 1 Introduction

The conventional livestock industry accounts for more than 18% of the greenhouse gas emissions that affect global climate change and is associated with various problems, such as environmental pollution, diseases, and animal ethics issues.^[^ ^1^, ^2^, ^3^ ^]^ Moreover, according to the Food and Agriculture Organization of the United Nations, meat consumption will increase by 14% by 2030 compared to the base period average of 2018–2020, driven largely by income and population growth.^[^ ^4^ ^]^ Because of these problems, the research interest and market for meat substitutes to replace real meat have been revitalized. Plant‐based meat substitutes have a limited ability to replace meat because their nutritional content and taste are very different from those of real meat.^[^ ^5^, ^6^, ^7^ ^]^ Therefore, in recent years, substantial research has been done on cultured meat, which is meat produced by culturing animal cells in vitro. However, just producing a collection of myotubes is insufficient in many aspects, especially in economic and productivity aspects. Quantitative research is needed to provide solutions to the growing global meat consumption and related problems. The present time calls for technology to produce steak‐type cultured meat with a realistic centimeter (cm) size.^[^ ^8^, ^9^ ^]^

Efforts to culture meat began with the production of muscle‐like protein for astronauts and space station residents.^[^ ^10^ ^]^ Since Mark Post and his research team introduced a hamburger patty produced by culturing muscle stem cells harvested from a cow in 2013, various types of cultured meat have been produced.^[^ ^5^, ^6^, ^7^, ^8^, ^9^, ^10^, ^11^, ^12^ ^]^ Recently, many studies have been focused on 3D cell culture for cultured meat.^[^ ^13^ ^]^ However, the large scale of 3D cell culture is still difficult, and development and optimization are in the early stage.^[^ ^14^ ^]^ There is a clear necessity for the convergence of large‐scale 3D cell culture and differentiation technology in a system for producing large‐scale 3D cell aggregates.

This study aimed to introduce 3D bioprinting as a system for producing large cell aggregates. 3D bioprinting is widely used in the field of tissue engineering for organ development for various applications, such as the restoration of soft tissues,^[^ ^15^ ^]^ human ears,^[^ ^16^ ^]^ and bones.^[^ ^17^ ^]^ By printing a computer‐modeled design with biomaterials and cells, it is possible to form a 3D structure and construct a large‐scale tissue. Bioinks for large‐sized cultured meat bioprinting should be composed of biocompatible components capable of supporting cells. Furthermore, a scaffold structure in which many cell aggregate of realistic size can be cultured to a stable structure is needed. This requires research on how to print scaffolds with high biocompatibility and excellent printability.^[^ ^18^ ^]^

In the study of cultured meat, it is important to study not only the scaffold, but also the cells constituting the cultured meat. To date, cells constituting cultured meat have been produced in three major ways. First, they can be produced by collecting a small amount of muscle stem cells and expanding the cells.^[^ ^19^, ^20^ ^]^ Second, somatic cells can be transformed into induced pluripotent stem cells and differentiated into muscle cells.^[^ ^21^ ^]^ Third, somatic cells can be directly transdifferentiated into muscle cells.^[^ ^22^, ^23^ ^]^ The first method is the most widely used. However, primary muscle satellite cells maintain differentiation capacity only within 10 passages and have limitations in proliferation capacity.^[^ ^24^ ^]^ Therefore, it is difficult to maintain a stable and constant cell population which requires the continuous collection of cells through biopsy from animals, posing a risk of causing continuous pain to animals. When using the second method, it is still controversial, such as immunogenicity, genome instability, or tumorigenicity.^[^ ^25^ ^]^ The third method, involving direct transdifferentiation, allows stable proliferation in a somatic cell state and differentiation control as long as the transdifferentiation conditions are properly set.^[^ ^26^ ^]^ Due to concerns about genetic manipulation by transgenesis and difficulties in establishing transdifferentiation conditions, the use of transdifferentiation has not yet been actively researched in cultured meat studies. Previous studies, by introducing genes such as *MyoD* and *PPARγ2* into naturally immortalized bovine fibroblasts, it was possible to obtain cell types capable of stably proliferating and differentiating into muscle and fat.^[^ ^27^, ^28^ ^]^ The cells were maintained as fibroblasts in a general growth medium (GM) without any additives and proliferated at a constant rate. When they were placed in a differentiation medium (DM), they directly differentiated into the specific desired cell types, muscle and fat.

In this study, after encapsulation in gelatin methacryloyl (GelMA) hydrogel and digital light processing‐based (DLP) 3D printing, cells were cultured in this structure to produce steak‐type cultured meat at the cm scale. The polymerized construct should have a porous structure and sufficient mechanical strength so as to not interfere with cell proliferation. For this purpose, the well‐known GelMA hydrogel was selected in this study.^[^ ^29^ ^]^ Owing to its capacity to maintain high cell stability and viability, GelMA, a natural biomaterial, is more suitable for producing cell‐containing bioinks.^[^ ^30^, ^31^, ^32^ ^]^ Printing a cm‐scale 3D structure with microchannels requires high printing accuracy and geometrically complex printability for normal cell culture. Therefore, we adopted DLP bioprinting. The DLP bioprinting technology achieves high‐speed printing of 3D hydrogels by using digital mask projection to induce photocrosslinking‐based polymerization with a print resolution ranging from 30 to 100 µm. It is used in many applications, including tissue engineering, biosensors, microchips, and drug delivery systems. High biocompatibility and precise printability were achieved through optimization using a new measurement method termed photorheological measurement. Finally, scaffolds with cells and large microchannels were successfully printed.

For sustainable and stable cell populations, myogenesis and adipogenesis were induced using naturally immortalized bovine embryonic fibroblast (BEFS) cells transformed with *MyoD* and *PPARγ2*, respectively, termed BEFS‐teton‐MyoD and BEFS‐PPAR*γ*2, respectively.^[^ ^28^ ^]^ To produce cultured meat, unlike in conventional differentiation methods that use isobutyl‐methylxanthine or dexamethasone, we used oleic acid, a fatty acid, to induce adipogenesis, based on previous research results.^[^ ^33^, ^34^ ^]^

**Figure 1:**
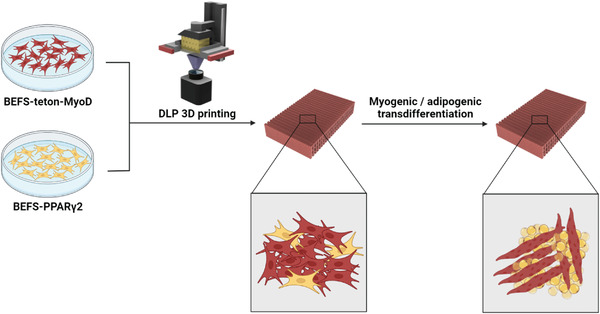
Schematic diagram of the 3D‐bioprinted steak‐type cultured meat production process. The method allows not only using a simple mixture of muscle and fat cells but also controlling the muscle‐to‐fat ratio.

To allow muscle and fat to be mixed for simultaneous culture, oleic acid was added to the myogenesis DM (MDM) to prepare a myogenesis/adipogenesis differentiation medium (MADM). Finally, the cells were mixed with GelMA hydrogel for DLP printing and proliferated, and myogenesis and adipogenesis were induced simply by changing the medium to obtain steak‐type cultured meat with an appropriate proportion of muscle and fat (**Figure** **1**). Besides allowing the use of a simple mixture of muscle and fat cells, this method enables controlling the muscle‐to‐fat ratio as desired. The possibility of using the steak‐type cultured meat produced by successful cell proliferation and differentiation was confirmed by preparing a simple fried dish with it.

## 2 Experimental Section

### 2.1 Materials

Tartrazine pigment was purchased from GreenTech (Daejeon, Gyeonggi, South Korea). A live/dead cell viability kit was purchased from Invitrogen (Carlsbad, CA, USA). Dulbecco's modified Eagle's medium (DMEM), penicillin streptomycin (p/s), fetal bovine serum (FBS), l‐glutamine, and phosphate‐buffered saline (PBS, pH 7.4) were purchased from WelGene (Daegu, Gyeongbuk, South Korea). Beef (tenderloin) was purchased from Local market (Emart, Gwamgjin‐Gu, Seoul). Gelatin (Type A, 300 bloom from porcine skin), methacrylic anhydride (MA), lithium phenyl‐2,4,6‐trimethylbenzoylphosphinate (LAP, ≥ 95%), doxycycline, insulin, oleic acid, Oil Red O staining solution, bovine serum albumin, and Triton X‐100 were purchased from Sigma‐Aldrich (St. Louis, MO, USA). All other chemical agents used in this study were of analytical grade.

#### 2.1.1 GelMA Synthesis

GelMA was synthesized as described previously.^[^ ^29^ ^]^ Briefly, 10% w/v type A porcine skin gelatin was added to PBS and stirred at 50 °C until fully dissolved. MA was then added at a rate of 1 mL min^−1^ to a final concentration of 15% v/v. The solution was allowed to react at 50 °C for 4 h. The reaction was stopped by adding 400% v/v PBS at 40 °C. Then, the mixture was dialyzed against distilled water for 1 week using a 12–14 kDa cutoff dialysis tubing to remove residual MA. Finally, the solution was lyophilized for 4 d to obtain fully dried pure GelMA (Figure S1a, Supporting Information).

### 2.2 Bioink Preparation

**Figure 2:**
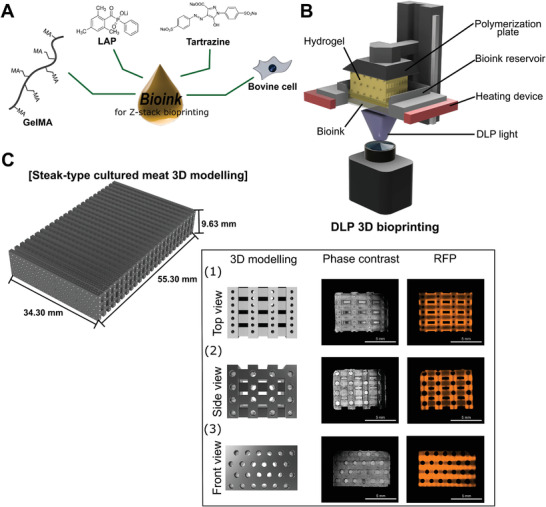
a) Schematic representation of the composition of the bioink including GelMA, LAP, tartrazine, and BEFS cells. b) Schematic representation of the DLP bioprinting process for fabricating structures using bioink. c) Schematic representation of steak‐type cultured meat 3D modeling (width, 34.3 mm, length, 55.3 mm, and height, 9.63 mm).

GelMA was synthesized as described previously.^[^ ^1^ ^]^ To prepare the bioink for DLP bioprinting, lyophilized GelMA was completely dissolved (10% w/v) in Dulbecco's (D)PBS at 37 °C containing 1% v/v p/s, 2% v/v) FBS, 0.5% w/v LAP, and 1% v/v tartrazine pigment. Cultured BEFS cells were detached from the culture plate and added to the GelMA solution at a concentration of 2 × 10^6^ cells mL^−1^ (**Figure** **2**a).

### 2.3 ^1^H Nuclear Magnetic Resonance (^1^H NMR) Spectroscopy

^1^H NMR spectroscopy was used to determine the degree of substitution of GelMA. Gelatin and GelMA were dissolved in D_2_O and analyzed using a 500 MHz FT (Fourier transform)‐NMR spectrometer (Varian; Palo Alto, CA, USA).

### 2.4 Modeling and Printing

An IM2 DLP 3D printer (Carima, Seoul, Korea) was used to print the BEFS cell‐laden 3D hydrogel construct to produce steak‐type cultured meat. The bioink reservoir was preheated to 37 °C and filled with 20 mL of bioink, and the optimal light exposure time was set. The UV–vis light (365–420 nm) intensity was set to 1.97 mW cm^−2^. Printing was carried out for a total of 96 layers by setting the focal layer to 100 µm. The printed steak‐type cultured meat hydrogel construct was carefully removed from the plate, washed with DPBS (Dulbecco's phosphate buffered saline) containing 1% p/s, and stored in cell culture medium (Figure 2b).

A steak‐type 3D model was created using Fusion 360 (Autodesk; San Rafael, CA, USA). The microchannels in the side part of the “steak” were designed to have a diameter of 800 µm, and the small microchannels between the top and bottom were designed to be 300 µm in diameter. The overall dimensions were 34.3 mm width, 55.3 mm length, and 9.63 mm height, and the total volume was 10 062.162 mm^3^. The stereolithography model was sliced ​​at 100 µm increments (Figure 2c).

### 2.5 Photorheological Measurement

Photorheology was performed using an HAKKE MARS 40 instrument (Thermo Fisher Scientific, Waltham, MA, USA) equipped with an accessory light module. To investigate the rheological behavior of the bioink when the DLP printer was irradiated with light, a light source with a wavelength similar to that of the DLP printer was generated using an OmniCure LX 505 (Lumen Dynamics; Mississauga, ON, Canada) equipped with a 405 nm light‐emitting diode (LED) channel. The light intensity was set at 2.45 mW cm^−2^ and irradiation were guided through the collimator and reflected toward the measuring glass (Figure S2a, Supporting Information). The LED intensity was measured using a rheometer measuring glass and set to the same intensity as that of the DLP printer. Bioink was loaded at 100 µm intervals between the glass and the bottom plate. The oscillation experiment was conducted with an oscillating shear strain of 0.008% at a frequency of 20 Hz. To investigate the relationship between *E*_min_ and *E*, photorheological measurements were made under the conditions of fixed depth of cure (*C*_d_) and height of gelation (*H*_d_) of the hydrogel. *E*_min_ is the minimum critical energy required for the gelation height of the hydrogel to reach the cure depth and was calculated by modifying the formula to explore the cure depth according to the light exposure energy^[^ ^35^ ^]^ as follows

(1) $$E_{\min}=E\times e^{\left(\frac{C_{d}}{H_{d}}\right)}$$

Horizontal and vertical printability analyses were performed to evaluate printability according to the light exposure time. First, a model was prepared to measure horizontal printability (Figure S2c, Supporting Information). The model was designed to have a horizontal microchannel (1000 × 1000 µm^2^) in the center. Based on the gelling time (20 s) obtained by photorheological measurement, in the model, the gelling time was increased up to 40 s in 5 s increments. Then, the area of the hollow space of the microchannel of the printed hydrogel was measured. The hollow space ratio was obtained by calculating the difference between the expected area of the hollow space (*M*_a_) based on modeling and the actual area of the hollow space (*P*_a_) obtained after actual printing.

(2) $$\mathrm{Hollow}\;\mathrm{space}\;\mathrm{ratio}\;\left(\%\right)=\frac{P_{a}}{M_{a}}\times 100\;\left(\%\right)$$

Second, a model was prepared to measure vertical printability (Figure S2d, Supporting Information). This model was designed to have microchannels with ten different diameters (50–500 µm). Based on the gelling time (20 s) obtained by photorheological measurement, in the model, the gelling time was increased up to 32 s in 3 s increments. Then, the establishment of microchannels in the printed hydrogel was confirmed. If a microchannel was blocked by more than 30%, it was considered not realized.

To evaluate biocompatibility according to the light exposure time, a live/dead assay was performed after three different exposure times (20, 30, and 40 s). Printed cell viability was investigated using a live/dead cell viability kit containing calcein AM (calcein acetoxymethy ester) and ethidium homodimer‐1. BEFS cells printed with GelMA hydrogel at 2 × 10^6^ cells mL^−1^ were exposed to light for different times (20, 30, and 40 s) and incubated in a humidified atmosphere of 5% CO_2_ at 37 °C. After 3 d of incubation, the samples were plated in a 24‐well plate and 500 µL of staining reagent was added to each well according to the manufacturer's protocol. Then, the samples were imaged using a Lionheart FX automated microscope (BioTek, Winooski, VT, USA). Cell viability was calculated as the proportion of live cells in the total number of cells (see Section 2.11).

### 2.6 Cell Culture and Differentiation

BEFS‐teton‐MyoD and BEFS‐PPAR*γ*2 cells were provided by Prof. Hong Gu Lee (Konkuk University, Seoul, Korea). All cells were maintained in a humidified atmosphere of 5% CO_2_ at 37 °C. All cells were grown in DMEM supplemented with 10% FBS, 1% p/s, and 2 mM l‐glutamine, as previously described.^[^ ^28^ ^]^

To induce myogenic differentiation, BEFS‐teton‐MyoD cells were plated and grown until 100% confluence was reached. Then, the GM was replaced with MDM (DMEM supplemented with 2% horse serum (HS; Gibco, Carlsbad, CA, USA), 1% p/s, 2 mM l‐glutamine, doxycycline (1 µg mL^−1^), and insulin (10 µg mL^−1^)), and the cells were cultured for 6 d. The DM was refreshed every 2 d.

To induce adipogenic differentiation, confluent BEFS‐PPAR*γ*2 cells were subjected to differentiation by replacing GM with MADM (DMEM supplemented with 10% FBS or 2% HS, 1% p/s, 2 mM l‐glutamine, 10 µg mL^−1^ insulin, doxycycline (0 and 1 µg mL^−1^), and oleic acid (0–0.5 mM)) and maintained for 2 d.

### 2.7 Oil Red O Staining

BEFS‐PPAR*γ*2 cells were washed with PBS three times and fixed with 4% paraformaldehyde at room temperature for 15 min. The fixed cells were washed with PBS three times and stained with 0.3% Oil Red O staining solution for 30 min. The samples were visualized using the Lionheart FX automated microscope. After three washes with PBS, the Oil Red O stain was eluted by adding 100% isopropanol for 15 min and the absorbance at 490 nm was measured using a GloMax Explorer Multimode Microplate Reader (Promega, Madison, WI, USA). All tests were performed in triplicate.

### 2.8 Quantitative Reverse Transcription (RT‐q)PCR Analysis

Total RNA was isolated from BEFS‐teton‐MyoD cells after 0, 1, 2, 4, and 6 d of differentiation using TRIzol reagent (iNtRON, Seongnam, Gyeonggi, Korea) according to the manufacturer's instructions. RNA quality was determined at an absorbance of 260 nm (A260) using a BioTek plate reader and Take3 plate (BioTek, Winooski, VT, USA). RNA quality was evaluated based on the A260/280 ratio. cDNA was synthesized from the total RNA using HKGscript 5X RT Premix (HK Genomics, Daejeon, Korea), the conditions were 50 °C for 15 min and 70 °C for 15 min. Real‐time quantitative PCR (qPCR) was run in a LightCycler 96 system (Roche, Basel, Switzerland), using SYBR Green I qPCR Master Mix (Genetbio, Daejeon, Korea). Primer sequences for the target genes were as follows: Glyceraldehyde‐3‐phosphate dehydrogenase (*GAPDH*) forward: 5′‐CAGTATGATTCCACCCACGGC‐3′, reverse: 5′‐ATCTCGCTCCTGGAAGATGGTG‐3′; *MyoD* forward: 5′‐CAACTGTTCCGACGGCATGATG‐3′, reverse: 5′‐CGCTGTAGTAAGTGCGGTCGTA‐3′; *MyoG* forward: 5′‐GGCGTGTAAGGTGTGTAAGAGG‐3′, reverse: 5′‐CCTGGAAGCCTTCATTCACCTT‐3′; Myosin heavy chain 1 *(MYH1)* forward: 5′‐GGCCAGACTGTAGAGCAGGTAT‐3′, reverse: 5′‐GGCAACCATCCACAGGAACATC‐3′. Thermal cycling conditions were: 95 °C for 10 min, followed by 40 cycles at 95 °C for 15 s, and 60 °C for 40 s.

### 2.9 Immunocytochemistry

Cells were fixed with 4% paraformaldehyde at room temperature for 15 min and washed with PBS three times. For permeabilization, the cells were incubated in 0.3% Triton X‐100 in PBS for 15 min. Then, the samples were blocked in 5% bovine serum albumin in 0.05% Triton X‐100 (prepared in PBS) for 1 h and treated with an anti‐*α* tubulin primary antibody (1:100 in PBS; Developmental Studies Hybridoma Bank, Iowa City, MA, USA) or anti‐MF20 primary antibody (1:200 in PBS; Developmental Studies Hybridoma Bank, Iowa City, MA, USA) at room temperature for 1 h. Subsequently, the samples were washed with PBS and stained with Alexa Fluor 488‐labeled secondary antibodies (1:300 in PBS; Invitrogen, Carlsbad, CA, USA) for 1 h and counterstained with 4,6‐diamidino‐2‐phenylindole dihydrochloride (DAPI) (300 nm, Invitrogen, Carlsbad, CA, USA). The samples were visualized under the Lionheart FX automated microscope. MYH‐positive cells were analyzed using the National Institutes of Health (NIH) ImageJ software.

### 2.10 CellTiter‐Glo Proliferation Assay

3D cell proliferation was assayed using CellTiter‐Glo 3D reagent (Promega, Madison, WI, USA) according to manufacturer's protocol, with slight modification. Cells were placed in 24‐well plates and washed with DPBS three times. CellTiter‐Glo 3D reagent was added to each well and the plate was incubated in a GloMax Explorer Multimode Microplate Reader under shaking for 30 min. Then, 100 µL of the mixtures was transferred to a white 96‐well plate and luminescence signals were measured using the GloMax Explorer Multimode Microplate Reader.

### 2.11 Live/Dead Assay

To investigate the cytotoxicity of GelMA, 3D‐printed cell viability was investigated using a live/dead cell viability kit containing calcein AM and ethidium homodimer‐1. Cells were printed with GelMA hydrogel at 2 × 10^6^ cells mL^−1^ and incubated in a humidified atmosphere of 5% CO_2_ at 37 °C. After 1, 3, 5, and 7 d of incubation, the cells were placed in 24‐well plates and 500 µL of staining reagent was added to each well according to the manufacturer's protocol. Then, the cells were imaged using the Lionheart FX automated microscope. Cell viability was calculated as the proportion of live cells in the total number of cells.

### 2.12 Mechanical Properties

A CT3 Texture analyzer (Brookfield; Toronto, ON, Canada) with a 4500 g load cell (Brookfield; Toronto, ON, Canada) in compression mode was used to measure the compressive strength of steak‐type cultured meat and beef (tenderloin). To determine the difference in strength, it was prepared in two states (raw and pan‐fried). For the measurement, samples having a diameter of 8 mm and a height of 3 mm were prepared using a biopsy punch with a diameter of 8 mm. A probe 12.7 mm in diameter was used to compress with a trigger load of 0.05 N at a test speed of 0.05 mm s^−1^. The compressive modulus was determined as the slope of the linear region corresponding to 5–15% strain.^[^ ^29^ ^]^

### 2.13 Statistical Analysis

Data are presented as means ± standard deviations (SDs), and means were compared using an unpaired Student's *t*‐test or one‐way analysis of variance (ANOVAs) followed by Tukey's tests, as apt. A *p*‐value < 0.05 was considered statistically significant. All analyses were performed using GraphPad Prism 8.0.2 (GraphPad Software; La Jolla, CA, USA).

## 3 Results and Discussion

### 3.1 ^1^H NMR Spectroscopy

Gelatin methacrylation was verified using ^1^H NMR. Compared with the spectrum of unmodified gelatin, the GelMA sample showed new functional groups, marked in yellow (I) and blue (III) in Figure S1a,b in the Supporting Information. The peaks at around 5.3 and 5.5 ppm were assigned to acrylic protons of the grafted methacryloyl group, and the peak at 1.9 ppm was attributed to the methyl group of the grafted methacryloyl group. There was a decrease in the intensity of the peak at 2.9–3.1 ppm, which was assigned to lysine methylene (marked as red in (II)). As lysine is the target site for the reaction, this result was used to determine the degree of methacrylation, which was estimated to be 81.4%.

### 3.2 GelMA Bioink Preparation for 3D Bioprinting

Bioink in this study was prepared by mixing GelMA, photoinitiator, photoabsorber, and BEFS cells in DPBS (Figure 2a). GelMA is a hydrogel‐forming polymer consisting of natural gelatin with a photoreactive methacrylate group introduced into its backbone.^[^ ^36^ ^]^ As for the photoinitiator LAP, when used at a concentration of 0.5%, it has been previously established that the free radicals produced during processing do not have a significant cytotoxic effect.^[^ ^37^ ^]^ Tartrazine, an edible pigment, was used as a photoabsorber (Figure S1c, Supporting Information). The prepared bioink was transferred into a preheated bioink reservoir and immersed, and then printed on a polymerization plate using DLP light (Figure 2b).

### 3.3 3D Modeling of a Steak‐Type Construct

To establish a 3D printed cm‐scale cultured meat product, a cm‐scale scaffold that can be fully filled with cells while maintaining structural rigidity was designed. Steak‐type 3D modeling introduces microchannels into the cm‐scale structure. Microchannels of ≥250 µm enhance cell survival because they allow the circulation of nutrients and metabolic wastes, while providing structural stability to the cm‐scale cultured meat through the buoyancy of the culture medium (Figure 2c).^[^ ^38^, ^39^, ^40^, ^41^ ^]^ However, the printing of such microchannels with µm‐scale diameters requires high precision and the ink must have high printability. In general, the printability of an ideal bioink for 3D printing can be increased through the use of hydrophobic crosslinking enhancers, additional mixing of synthetic polymers, and additional use of photoinitiators, but this lowers their biocompatibility due to a poor cell culture environment.^[^ ^18^ ^]^ Optimization strategies are needed to improve printability and biocompatibility.

### 3.4 Optimization of the Light Exposure Time Based on Photorheometry

Hydrogel photopolymerization by DLP bioprinting is affected by various variables, such as the printing speed, light intensity, light exposure time, and wavelength. Several parameters, including the mechanical speed, light intensity, light exposure time, and wavelength were fixed to establish the optimal polymerization conditions to print microcontrollable structures. The problem of mismatch between the DLP light wavelength and the absorbance of the prepolymer solution was solved by using a yellow photoabsorber^[^ ^42^ ^]^ (Figure S1d, Supporting Information). The DLP light intensity was set to the lowest intensity possible for printing (1.97 mW cm^−2^) to minimize cell stress.

**Figure 3:**
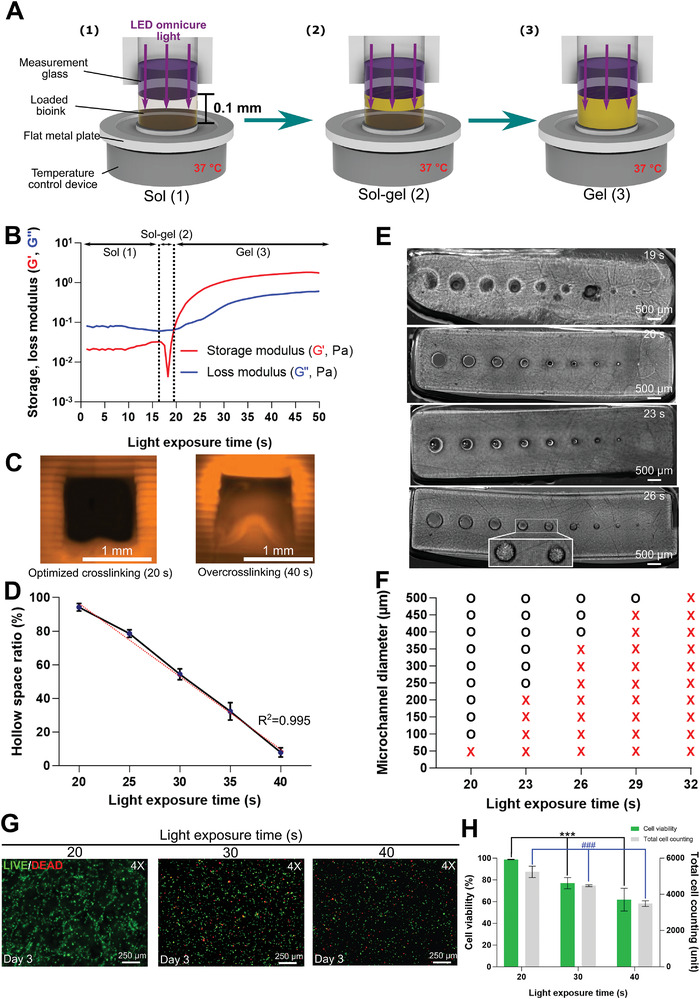
a) Schematic diagram of the photorheological measurement process. b) Photorheological measurement result of the bioink. c) Printed horizontal printability analysis of constructs exposed to light for different times (20 and 40 s). d) Hollow space ratio graph in function of light exposure time. Data are means ± SDs, *n* = 3. e) Printed vertical printability analysis of constructs exposed to light for different times (19, 20, 23, and 26 s). f) Vertical microchannel diameter in function of light exposure time. Xs indicate microchannels blocked for >30%. g) Fluorescence microscopy images of BEFS cells on day 3 in function of light exposure time (20, 30, and 40 s) in a live/dead assay. h) Cell viability and total cell counts obtained by the live/dead assay in function of light exposure time (20, 30, and 40 s). Data are means ± SDs (*n* = 3; ***p < 0.001) and were analyzed by one‐way ANOVA followed by Tukey's post hoc tests.

In 3D bioprinting of steak‐type cultured meat, it is important not only to have optimal printability for printing the complex steak‐type structure with microchannels, but also to maintain high cell viability after printing. Since DLP printing forms a hydrogel based on photocrosslinking, excessive light exposure induces undesirable crosslinking and scattering, resulting in over‐crosslinking in regions beyond the focal layer.^[^ ^43^ ^]^ It is possible to optimize the printability and cell viability by printing at a minimal light energy. Since the light intensity of the DLP printer was fixed at the minimum intensity, the light energy was adjusted by adjusting the light exposure time.^[^ ^44^, ^45^ ^]^ We used photorheological measurements to reproduce the height (*C*_d_) corresponding to 1 layer (= 100 µm) during 3D printing (**Figure** **3**aI). During the photorheological measurements, the bioink formed a hydrogel in a three‐step process (sol, sol–gel, and gel steps). The bioink loaded at 100 µm intervals was irradiated with LED light and subjected to the oscillation experiment. Scattering of the exposed light was inhibited by the photoabsorber. The photoinitiator was activated by the absorbed light energy to initiate free‐radical‐based polymerization. At the beginning of the measurement, the loss modulus (*G*″) of the bioink was measured to be larger than the storage modulus (*G*′), indicating that it was in a sol state. The bioink maintained the sol state because of the low viscosity of the solution used in the rheological measurement for a light exposure time of 17.47 s (Figure 3a,bI). With continuous light exposure, the bioink underwent sol–gel transition. It is assumed that the rapid decrease in *G*′ observed at this stage is due to the formation of a heterogeneous polymer because of the hybridization of the sol state and the gel state, which makes it difficult to provide accurate measurement values for the rheometer.^[^ ^46^, ^47^ ^]^ The rapid increase in *G*′ results in a *G*′–*G*″ intersection (*G*′ = *G*″), which is defined as the gelling time. It is also the gelation threshold to reach *E*_min_ to form a hydrogel. In conclusion, a 100 µm *H*_d_ was formed immediately after reaching the gelation threshold at which both the viscous and elastic forces started to rise. The light exposure time at this point was 19.56 s (Figure 3a,bII). Thereafter, as the hydrogel was further photocrosslinked, the viscoelasticity continued to increase (Figure 3a,bIII). The *E*_min_ required for the formation of a 100 µm high hydrogel was observed at the gelation threshold of 19.56 s, which was therefore considered to be the optimal light exposure time.

To confirm the printability at the optimized light exposure time obtained through photorheometry, we tested printability according to the light exposure time. First, the printability of the *Z*‐axis was confirmed through a horizontal analysis construct with a square‐shaped microchannel (Figure S2c, Supporting Information). At the optimized light exposure time (20 s), 94.21% ± 2.26% of the designed hollow space was realized; the hollow space was successfully printed (Figure 3c). Upon exposure for 25 s, the hollow space formed was 78.54% ± 2.27% of the designed space. Microchannels were formed, but were partially blocked. At 40 s, the longest exposure time tested, the hollow space was only 7.84% ± 2.82%, with most microchannels being blocked (Figure 3c,d). Second, the printability of the *X*–*Y* plane was assessed using a vertical construct with round‐shaped microchannels (Figure S2d, Supporting Information). At a light exposure time of 20 s or less, a measurable construct was not printed. At the optimized light exposure time, all microchannels, except for those with a diameter of 50 µm, were realized. A microchannel was considered to be blocked when less than 70% of the hollow space was realized. Upon exposure for 23 s, 50–200 µm microchannels were blocked, whereas 250–500 µm microchannels were formed successfully (Figure 3e,f). Data for other exposure times are shown in Figure 3f. Exposure for longer than the optimal time added unnecessary light energy, causing overcrosslinking and excessive light scattering. In particular, overcrosslinking hampered the formation of microchannels. Therefore, to successfully print steak‐type 3D cultured meat, one should optimize the light exposure time through photorheological measurement to optimize printability.

Cell viability upon exposure to light above *E*_min_ for three exposure times (20, 30, and 40 s) was assessed by the live/dead assay. Except for the light exposure time, all conditions were the same during the printing process (Figure 3g). The 20 s exposure group showed excellent cell viability. The cell viability after 20 s of exposure was 98.69% ± 0.26%. Cell viability in the 30 and 40 s exposure groups was 78.45% ± 3.32% and 52.92% ± 4.56%, respectively. Apart from the decrease in viability with increasing exposure time, proliferation decreased as indicated by a decrease in total cell count (Figure 3h). Thus, the longer the light exposure time, the worse the cell viability. These data indicated that the light exposure time should be optimized to optimize biocompatibility.

### 3.5 Establishment of Conditions for Myogenesis/Adipogenesis of Bovine Fibroblasts

**Figure 4:**
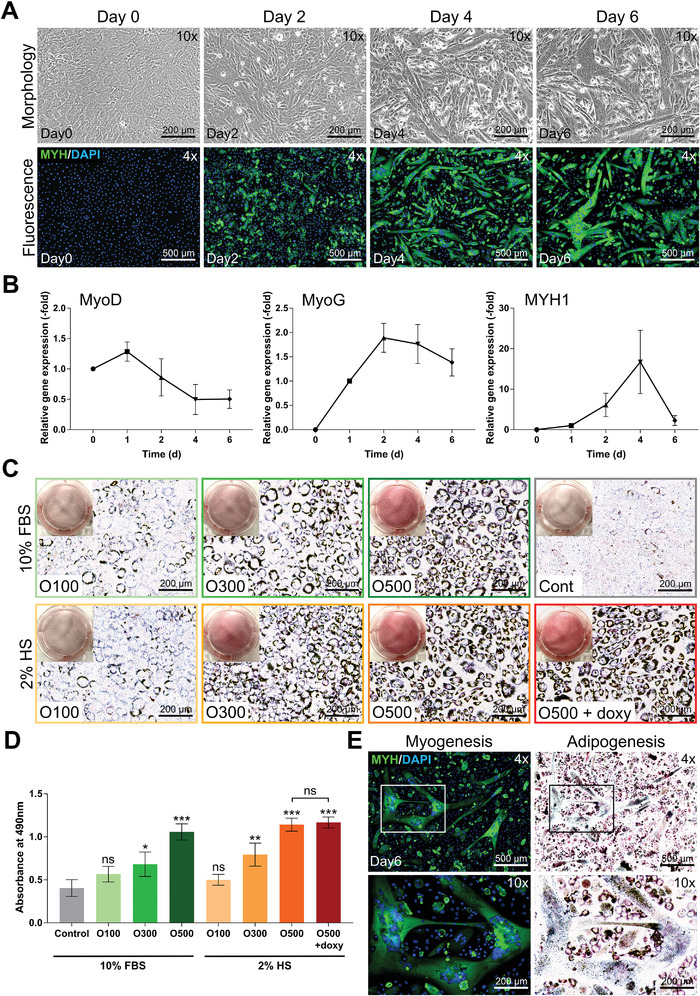
Myogenesis of BEFS‐teton‐MyoD cells in 2D and adipogenesis of BEFS‐PPAR*γ*2 cells using a fatty acid in our fibroblast transdifferentiation method. a) Morphology and immunofluorescence image of BEFS‐teton‐MyoD cells. The cells were differentiated for 6 d. The cells were immunostained for myosin heavy chain (MYH, green) and nuclei were stained with DAPI (blue). Scale bars = 200 µm (upper panels) and 500 µm (lower panels). b) RT‐qPCR analysis of BEFS‐teton‐MyoD cells differentiated for 0, 2, 4, and 6 d. Data are means ± SDs, *n* = 3. c) Oil Red O staining of BEFS‐PPAR*γ*2 cells in on various conditions. Scale bar = 200 µm. d) Oil Red O staining was quantified by measuring the absorbance at 490 nm. e) Immunofluorescence image of simultaneous myogenesis and adipogenesis. The cells were immunostained for MYH (green) and nuclei were stained with DAPI (blue). Scale bars = 500 µm (upper panel) and 200 µm (lower panels). Quantitative data are means ± SDs (*n* = 4). *p < 0.05; **p < 0.01; ***p < 0.001; ns, not significant, Student's *t*‐test.

To induce myogenesis in BEFS‐teton‐MyoD cells, BEFS‐teton‐MyoD cells were placed in MDM consisting of 2% HS, doxycycline, and insulin when they reached 100% confluence, based on previously reported differentiation conditions.^[^ ^28^ ^]^ After differentiation for 6 d with medium exchanges every 2 d, the myotubes gradually matured and grew longer and larger (**Figure** **4**a). *MyoD* gene expression increased on day 1, but decreased thereafter. *MyoG* and *MYH1* expression increased until day 2 and day 4, respectively, and then decreased (Figure 4b). This suggests that myogenesis induced by MyoD overexpression induced by doxycycline occurred until the myotubes matured.^[^ ^48^ ^]^ MYH1 RNA level decreased on day 6, but remained 2.26 times higher than on day 1. In addition, the protein expression was high on day 6 (Figure 4a), indicating that although the rate of increase in RNA level of MYH1 decreases, the total protein of myosin heavy chain, including subunits, is continuously expressed. The MYH‐positive cell areas were quantified from fluorescence images (Figure S3, Supporting Information).^[^ ^49^ ^]^

Next, we optimized the mixture of muscle and fat cells to establish conditions enabling efficient adipogenesis in BEFS‐PPAR*γ*2 cells under MDM conditions for myogenesis. To produce cultured meat, we used oleic acid instead of known fat differentiation drugs. We assessed the degree of adipogenesis using oleic acid at concentrations of 100, 300, and 500 nM with 10% FBS or 2% HS. Oil Red O staining (Figure 4c,d) revealed that for both 10% FBS and 2% HS, oleic acid at 500 nM induced better adipogenesis than 100 and 300 nM. In addition, the adipogenic efficiency was slightly better when 500 nM of oleic acid was added to 2% HS than when it was added to 10% FBS, and adipogenesis was not reduced even when doxycycline was added to 2% HS, as in the MDM condition. Therefore, MADM that can effectively induce adipogenesis was prepared by adding oleic acid to the MDM used to induce myogenesis in this study. Although the conventional adipogenesis protocols using drugs to induce adipogenesis require at least 6 d,^[^ ^50^ ^]^ in this study, using a high concentration of fatty acid, it was possible to rapidly induce adipocytes in 2 d.^[^ ^51^ ^]^

Finally, after growing BEFS‐teton‐MyoD and BEFS‐PPAR*γ*2 cells at a ratio of 8:2, differentiation into muscle and fat was induced using MADM. As myogenesis was induced in 6 d and adipogenesis in 2 d, after induction of the myotubes with MDM for 4 d, it was changed to MADM to induce myogenesis/adipogenesis simultaneously for the remaining 2 d (Figure 4e). As a result, myotubes and adipocytes were grown in a single space at the same time. Two days of exposure to oleic acid also seems induce accumulation of fat within myotubes. This phenomenon is consistent with in vivo skeletal muscle fat accumulation upon demands such as intramuscular fat formation after damages or other factors.^[^ ^52^ ^]^ In meat production, fat distribution within skeletal muscle is positively correlated with meat quality including flavor and juiciness.^[^ ^53^ ^]^ Thus, we successfully established a system that can induce fibroblasts into myotubes and adipocytes simultaneously.

### 3.6 Viability of BEFS‐MyoD and BEFS‐PPARγ2 Cells Encapculated in GelMA

**Figure 5:**
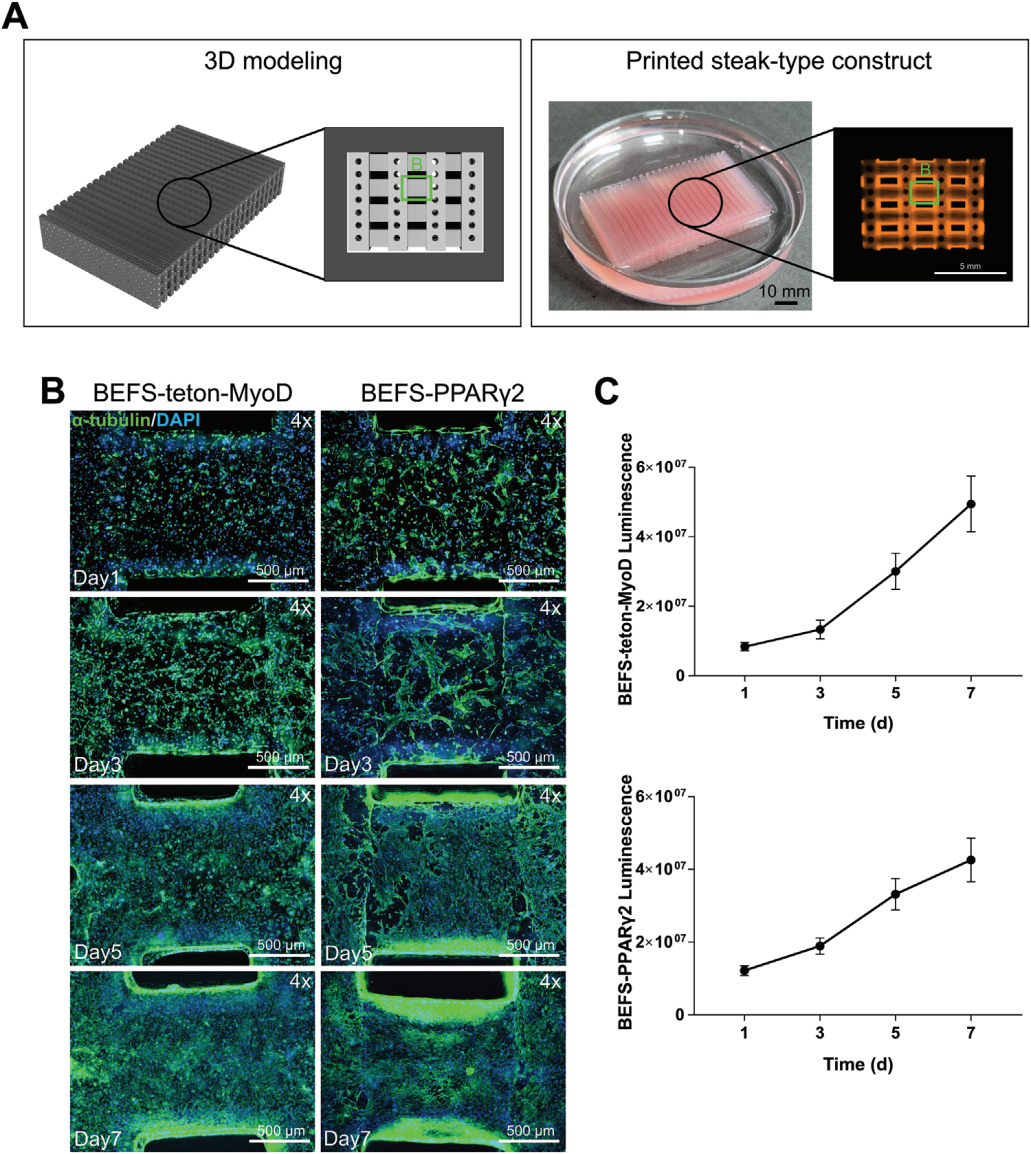
a) 3D design (left) of the steak‐type cultured meat and a photograph of printed steak‐type cultured meat (right). The internal structure was composed of microchannels. b) Immunofluorescence images of BEFS‐teton‐MyoD and BEFS‐PPAR*γ2* cells grown on GelMA scaffold for 1, 3, 5, and 7 d. The images acquired are magnifications of areas B (“leg” part) indicated in Figure 5a. Cells were immunostained for *α*‐tubulin (green) and nuclei were stained with DAPI (blue). Scale bars = 500 µm. c) Cell proliferation of BEFS‐teton‐MyoD and BEFS‐PPAR*γ*2 cells after 1, 3, 5, and 7 d measured using the CellTiter‐Glo 3D assay. Quantitative data are means ± SDs (*n* = 4).

We aimed at producing steak‐type cultured meat that has a diameter of a few centimeters, which is not easy to observe under a microscope. Therefore, for cell experiments and imaging, we produced slices of the steak‐type cultured meat structure. It was possible to identify the microchannels when the internal structure of the steak‐type cultured meat was enlarged (**Figure** **5**a).

To identify the biological effects of GelMA hydrogel, BEFS‐teton‐MyoD, and BEFS‐PPAR*γ*2 cells were encapsulated in GelMA hydrogel for 3D printing. Unlike in 2D, in a 3D environment, it is difficult to visually distinguish the time point at which cells reach 100% confluency. Therefore, the time point at which cells reached saturated confluency was first assessed by immunostaining and proliferation assays. DAPI/*α*‐tubulin staining images were used to confirm the proliferation of 3D‐printed cells (Figure 5b). When the cells were grown for 1, 3, 5, and 7 d after 3D printing, both cell types grew rapidly and reached nearly 100% confluence on day 7. As seen in fluorescence images, cells proliferated rapidly on the side adjacent to the microchannel, suggesting that cell proliferation was limited by the rate of media exchange through the microchannels. Therefore, in order to maximize cell proliferation, research on microchannel optimization such as size, number, and arrangement considering media perfusion will be needed in the future.

**Figure 6:**
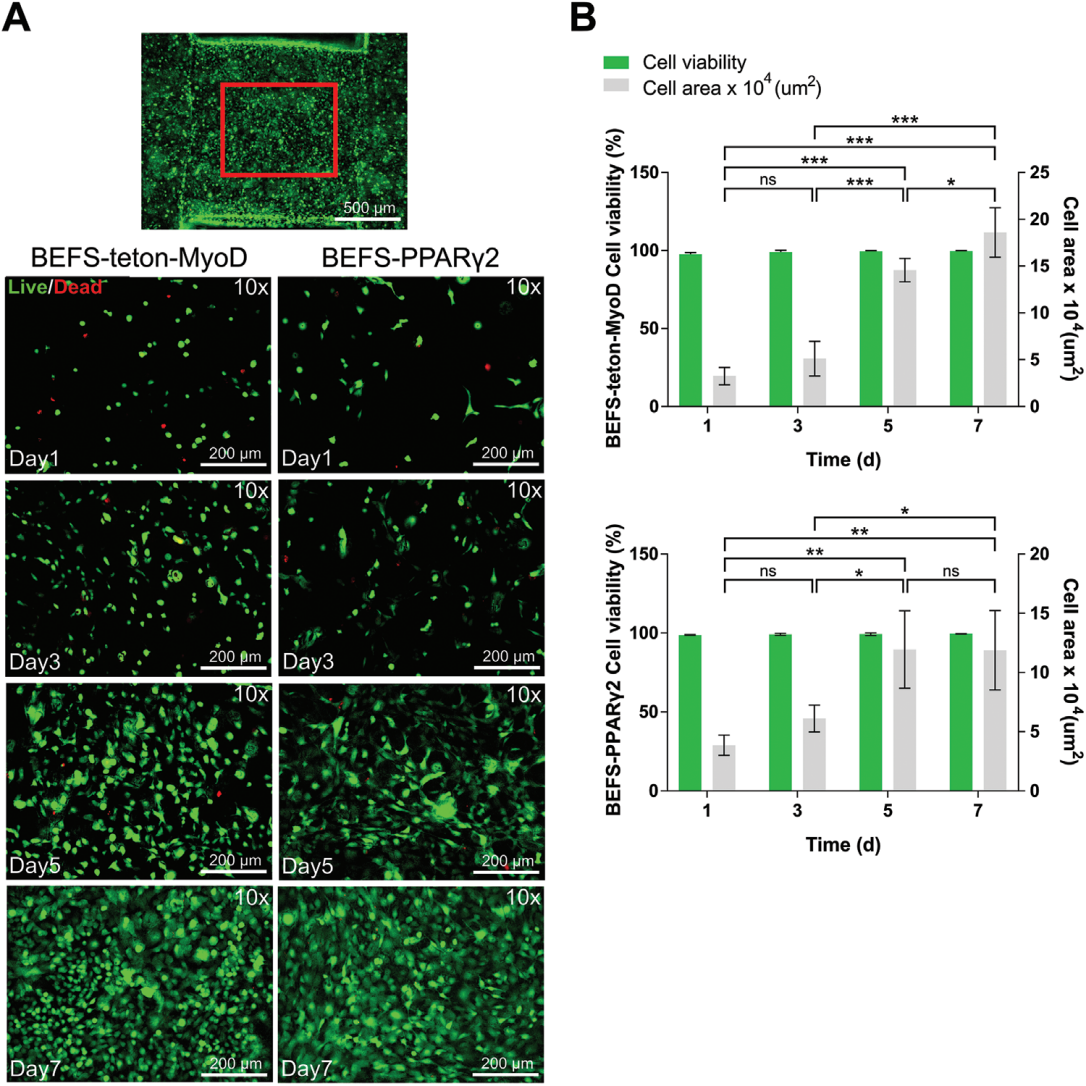
a) Live (green)/dead (red) staining assay of BEFS‐teton‐MyoD and BEFS‐PPAR*γ*2 cells grown in GelMA scaffold for 1, 3, 5, and 7 d. Scale bars = 200 µm b) Cell viability and cell area obtained from live/dead assays at 1, 3, 5, and 7 d. Data are means ± SDs (*n* = 4; *p < 0.05; **p < 0.01; ***p < 0.001; ns, not significant) and were analyzed by one‐way ANOVA followed by Tukey's post hoc tests.

Luminescence‐based quantification of the cells revealed that both cell types showed steady growth up to day 7 (Figure 5c). To check cell viability on day 7, cell viability was measured on days 1, 3, 5, and 7 by a live/dead assay (**Figure** **6**a,b). The results showed high cell viability (>95%) as both cell types proliferated up to 7 d after printing, indicating the safety of DLP printing using GelMA hydrogel. These results suggested that GelMA hydrogel provides an appropriate environment for cell proliferation.

### 3.7 Myogenesis/Adipogenesis of 3D‐Bioprinted BEFS‐MyoD Cells

When myocytes differentiate into muscle, adjacent cells fuse to form multinucleated cells, which develop into mature myotubes, in which nuclei as well as cells are aligned.^[^ ^54^, ^55^ ^]^ However, in 2D culture, when BEFS‐teton‐MyoD cells were transdifferentiated into myocytes and developed into myotubes, the nuclei fused to become multinucleated cells, and MYH, the myotube marker, was expressed, but no cell alignment was observed. To solve the problem of cell alignment, we assessed whether cell alignment was possible in DLP cell printing based on previous studies arguing that cell alignment is possible in extrusion‐type cell printing using hydrogels.^[^ ^56^ ^]^

**Figure 7:**
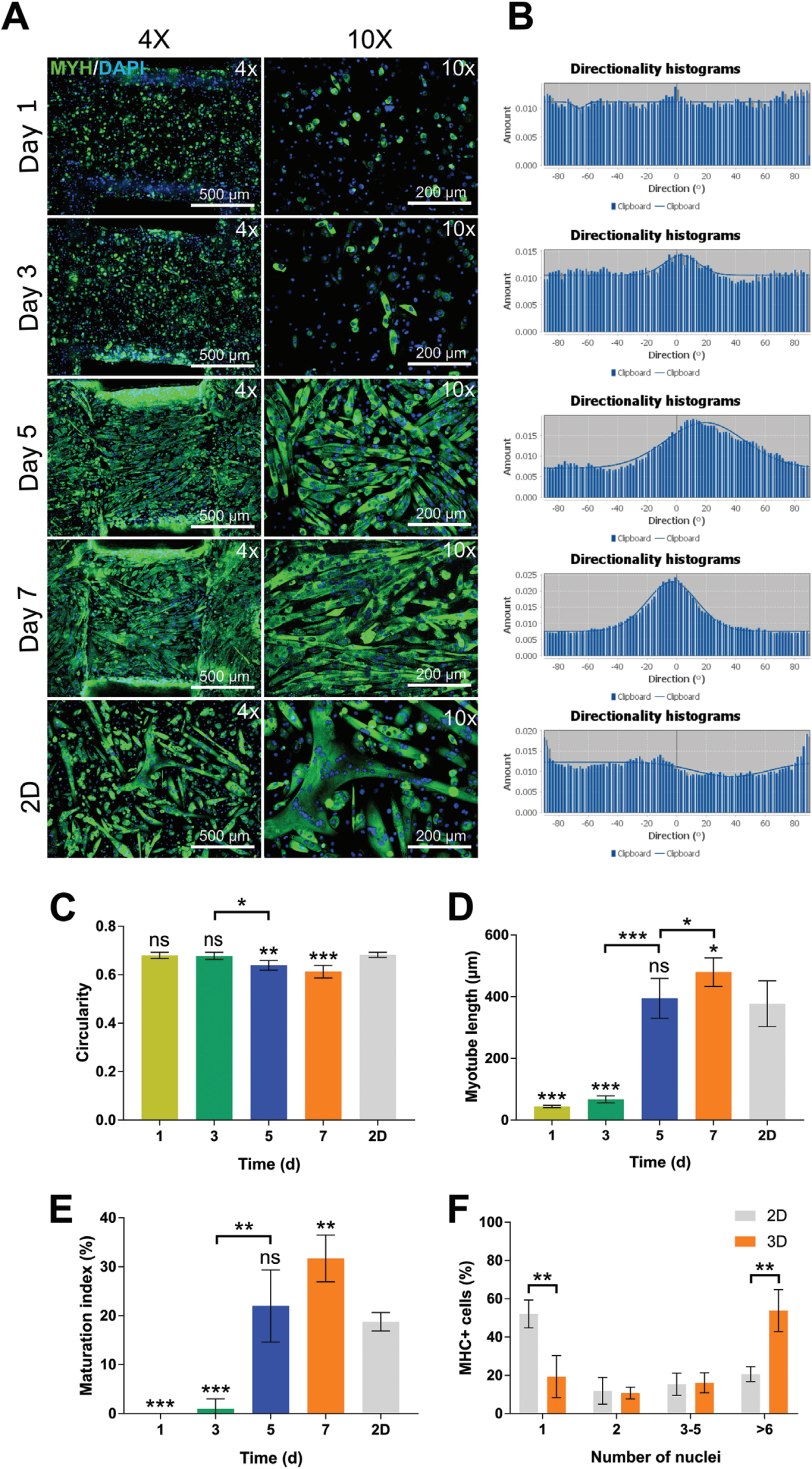
a) Immunofluorescence images of BEFS‐teton‐MyoD cells transdifferentiated after 1, 3, 5, and 7 d of proliferation. 2D‐cultured cells were differentiated at 100% confluence. Cells were immunostained for MYH (green) and nuclei were stained with DAPI (blue). Scale bars = 500 µm (upper panels) and 200 µm (lower panels). b) Histogram of BEFS‐teton‐MyoD cells directionality from (a) using the directionality tool in Fiji/ImageJ. c) Circularity value, d) myotube length, e) maturation index, and f) MYH‐positive cells measured from immunofluorescence images. Data are means ± SDs (*n* = 4; *p < 0.05; **p < 0.01; ***p < 0.001; ns, not significant) and were analyzed by one‐way ANOVA followed by Tukey's post hoc tests.

We first examined whether alignment upon differentiation into muscle was more effective for BEFS‐teton‐MyoD cells grown in a 3D environment than for those grown in a 2D environment. In the 3D environment, cells were grown and differentiated for 1, 3, 5, and 7 d, and in the 2D environment, cells were differentiated when they showed 100% confluence, followed by staining for MYH to confirm cell morphology (**Figure** **7**a). Until day 3, the cells showed low confluence, with few cells being fused and showing low MYH expression. On day 5, cell fusion and elongation started to be observed, and on day 7, cell density as well as length increased. However, in 2D, even when differentiated at 100% confluence, cell density was low, with highly variable cell morphology. The directional evaluation results of the image in Figure 7a are shown in Figure 7b. Distributions of BEFS‐teton‐MyoD cells orientation were quantified using the directionality tool in Fiji/ImageJ software, with a directionality histogram ranging between ±90°. In a 3D environment, directionality increased as the number of days increased, but no direction was shown in the 2D environment. By analyzing immunofluorescence images in four aspects, i.e., 1) circularity (Figure 7c); 2) myotube length (Figure 7d); 3) maturation index, i.e., the number of myotubes with more than five nuclei and the distribution of nuclei numbers in a myotube (Figure 7e); 4) distribution of MYH‐positive cell number (Figure 7f), we quantitatively examined myotube formation on each day in 2D and 3D cultures. According to the results, the myotube length and maturation index were the highest on day 7 in 3D hydrogel, with the lowest nuclear circularity. In the 2D culture, myotube length was similar to that in the 3D hydrogel on day 5, but the circularity was as high as that of 3D hydrogels on day 1 and 3. Comparison of the distribution of myotubes by nuclei number on day 7 of 2D culture and 3D hydrogel revealed that the number of MYH‐positive cells having only one nucleus was substantially higher in 2D culture, whereas the number of MYH‐positive cells having six or more nuclei was significantly higher in 3D hydrogel culture. Thus, GelMA hydrogel structures obtained by DLP printing more effectively induce myotube alignment than 2D culture environments.

**Figure 8:**
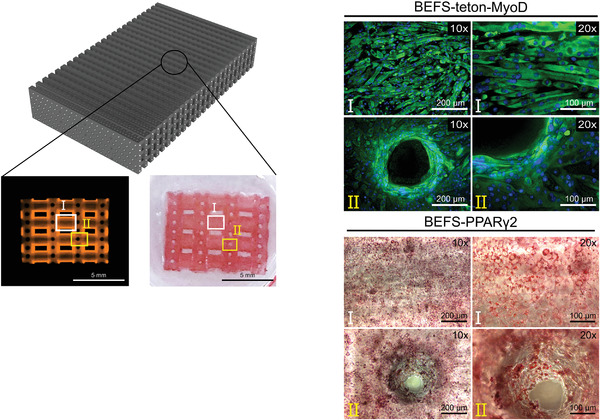
Images acquired after 3D printing and differentiation of BEFS‐teton‐MyoD and BEFS‐PPAR*γ*2 cells, respectively, followed by immunofluorescence and Oil Red O staining. In the 3D microstructure, the microchannel (I) and (II) were enlarged, respectively. Cells were immunostained for MYH (green) and nuclei were stained by DAPI (blue). Scale bars = 200 µm (left) and 100 µm (right).

To confirm that myogenesis and adipogenesis occurred in the leg and round parts (**Figure** **8**, panels I and II) of the GelMA hydrogel structure, after the printing of BEFS‐teton‐MyoD and BEFS‐PPAR*γ*2 cells with GelMA hydrogel and proliferation for up to 7 d, myogenesis and adipogenesis were induced, respectively, followed by immunofluorescence and Oil Red O staining (Figure 8). The results confirmed that the myotube was aligned in a straight line in the leg part and along the circle in the circular part (Video S1 (4 ×) and 2 (10 ×), *z*‐stack image from microchannel, Supporting Information). Oil Red O staining images showed that adipogenesis was effectively induced in both the leg and round parts. Together, our results indicated that the 3D environment in which cells were printed by DLP printing induced higher cell density and myotube alignment and fusion than the 2D environment and effectively induced adipogenesis.

### 3.8 Combination of Myogenic/Adipogenic Transdifferentiation in 3D Culture

**Figure 9:**
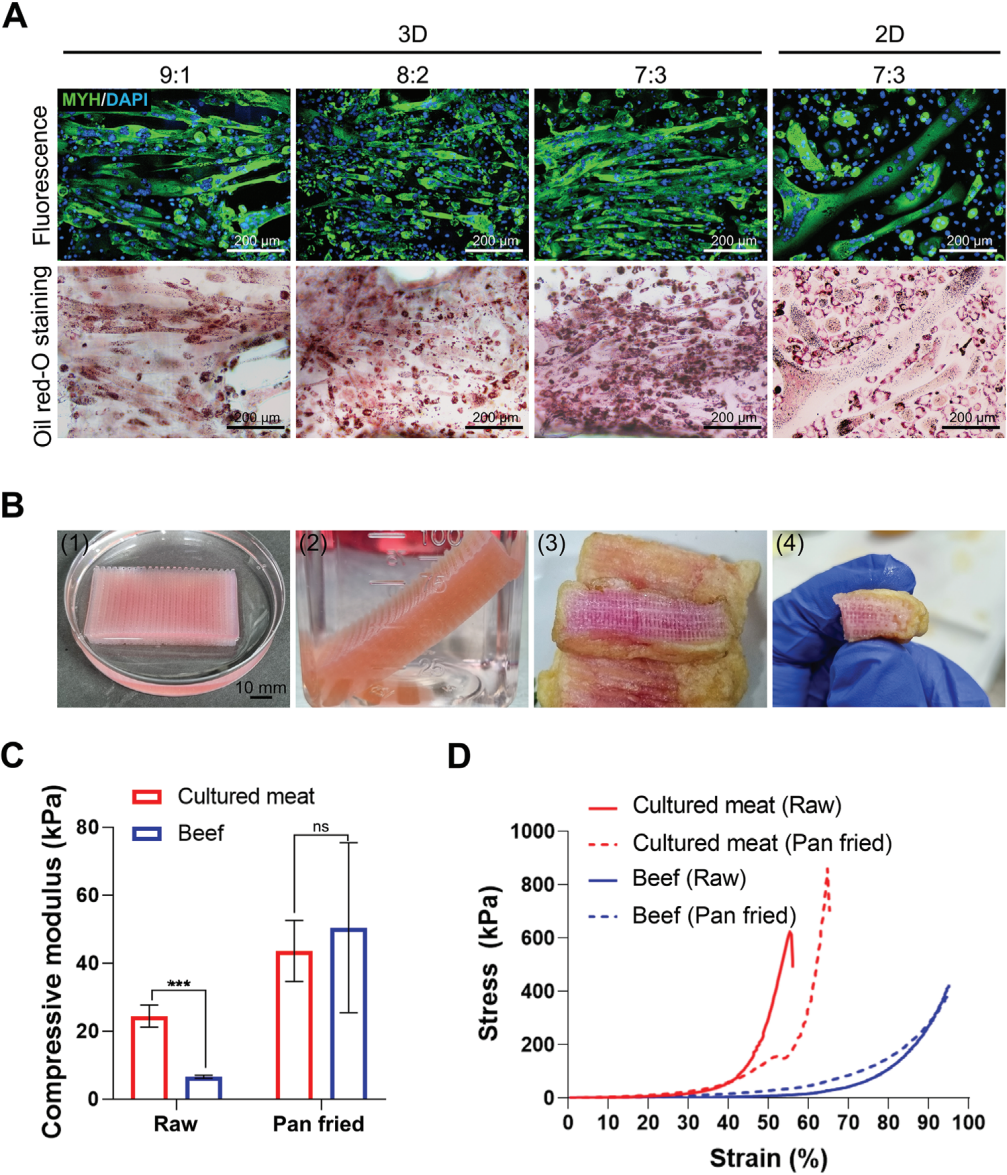
a) Immunofluorescence images and Oil Red O staining images of transdifferentiated myotubes and adipocytes. BEFS‐teton‐MyoD and BEFS‐PPAR*γ*2 cells were mixed at ratios of 9:1, 8:2, and 7:3. 2D culture was performed at a 7:3 ratio. Cells were immunostained for MYH (green) and nuclei were stained with DAPI (blue). Scale bar = 200 µm. b) Photographs of steak‐type cultured meat (1, 2) and the cross‐section (3, 4). Scale bar = 10 mm. c) Compressive modulus of steak‐type cultured meat and beef according to cooking: raw, pan‐fried. Data are means ± SDs (*n* = 6). ***p < 0.001; ns, not significant, Student's *t*‐test. d) Representative stress–strain curves of steak‐type cultured meat and beef according to cooking: raw, pan‐fried.

To obtain a mixture of muscle and fat like in real meat, the GelMA hydrogel was prepared with mixtures of muscle and fat cells at ratios of 9:1, 8:2, and 7:3. As in the above results, all hydrogels induced myogenesis and adipogenesis after growing for 7 d. On day 7, the MDM was changed to induce myogenesis for 4 d, and then it was changed to MADM to induce both myogenesis and adipogenesis. **Figure** **9**a shows the results of immunofluorescence staining and Oil Red O staining after mixing and differentiating cells at 9:1, 8:2, and 7:3 in 3D and at 7:3 in 2D. As indicated by the images, when adipocytes were mixed in at 10%, 20%, and 30%, myogenesis and adipogenesis were effectively induced.

Finally, a steak‐type hydrogel construct (34.30 mm width × 55.30 mm length × 9.63 mm height) was designed to prepare cultured meat of a size that can be actually sampled, and cell printing was performed after encapsulating BEFS‐teton‐MyoD cells (Figure 9b). After 4 weeks of culture, it was differentiated into myotubes using the above protocol. The cultured meat was deep‐fried after being coated with frying powder. In the frying powder coat, the cultured meat maintained its size and shape nearly perfectly, and even when cut with a knife, it maintained its shape without collapsing. Thus, we successfully reproduced a mixture of fat and muscle in 3D to simulate real meat. By manufacturing steak‐type cultured meat at the cm scale, the possibility of mixing fat to enhance the flavor of the cultured meat and scaling‐up of production was confirmed.

### 3.9 Mechanical Analysis

Compressive modulus was measured to compare the texture characteristics of steak‐type cultured meat with that of beef (tenderloin). As a result, the raw beef showed a relatively low modulus (6.55 ± 0.48 kPa). On the other hand, the raw cultured meat (Figure S5a, Supporting Information) had mechanical rigidity due to the stable structure of the printed GelMA hydrogel with a strength of 24.43 ± 3.29 kPa. However, the pan‐fried beef samples showed a high strength value of 50.411 ± 25.01 kPa, and a large change in mechanical stiffness was observed. In the process of obtaining a sample using a biopsy punch, a large deviation occurred due to the nonhomogeneous fat and muscle composition of beef.^[^ ^57^ ^]^ Similarly, changes in mechanical stiffness were also observed in pan‐fried cultured meat samples (Figure S5b, Supporting Information), and the compressive modulus was 43.59 ± 8.99 kPa. Relatively limited variation occurred due to the composition of homogeneous components and there was no statistically significant difference with pan‐fried beef samples. Both beef and cultured meat increased mechanical stiffness after cooking, and it was found that pan‐fried beef and cultured meat had similar compressive strength (Figure 9c). In strain‐stress curves, there were significant differences between beef and cultured meat. In raw and pan‐fried beef, the yield point did not occur, and there was no difference due to cooking. On the other hand, in the case of raw cultured meat, the yield point occurred at 55.33% strain showing the lowest elastic force among the experimental groups. In the case of pan‐fried cultured meat, the yield point was observed at 64.67% strain. It was confirmed that the elasticity of 3D printed cultured meat increased through the cooking process (Figure 9d). Although it was difficult to exhibit the same texture and mechanical rigidity as meat, hydrogels containing high moisture content proved to be eligible for cooking through a large amount of cell proliferation and differentiation (Videos S3–S5, Supporting Information).

## 4 Conclusion

To produce cultured meat hydrogel at the cm scale, this study aimed to develop ways to optimize bioink using photocrosslinkable materials for DLP printing and to demonstrate the feasibility of producing cultured meat by differentiating fibroblasts into muscle and fat cells followed by proliferation. By using a tartrazine as a photoabsorber^[^ ^42^ ^]^ in the bioink using GelMA as a homopolymer, it was synchronized with the light wavelength band of the DLP printer. The limitations of the cm‐scale hydrogel production due to the mechanical properties of GelMA were solved through 3D modeling by introducing microchannels and using buoyancy. Photorheological measurements can be used to investigate the minimum and optimal light exposure time and energy for the bioink for easy optimization of the printing speed, printability, and cell viability. This study achieved practical optimization of bioink and showed that printed scaffold biofabrication, which is ideal for cultured meat research, is possible.^[^ ^18^ ^]^

We adopted a method of transdifferentiating naturally immortalized fibroblasts rather than using primary cells, which have difficulties in maintaining stemness in the production of muscle cells and adipocytes. Further, this has great advantages over primary cells in terms of animal ethics and cost, and it is fast and technically simple, suggesting the high potential of this method for scaling‐up and process minimization for mass production of cultured meat in future (Figure S4, Supporting Information). In previous studies on cultured meat, fat, muscle, or blood vessels were cultured separately and assembled into a single mass,^[^ ^58^ ^]^ but in this study, fibroblasts were found to be capable of differentiating into muscle and fat cells and were simultaneously printed in one model to induce transdifferentiation into muscle and fat, allowing control of the muscle‐to‐fat ratio. However, transdifferentiation method using doxycycline is toxic and unfavorable for food application. For food safety, inducible systems using doxycycline can be replaced with nontoxic substances such as cumate.^[^ ^59^ ^]^ In addition, alternative transdifferentiation method using mRNA delivery system or plasmid transfection method also can be investigated for food application.^[^ ^60^, ^61^ ^]^ Furthermore, in this study, the use of photocrosslinking and animal‐based materials such as gelatin, FBS could not be completely excluded. To solve the safety and ethical problems, there is a need to conduct research on plant based polymers^[^ ^62^ ^]^ (zein, soy) and FBS substitution^[^ ^63^ ^]^ as well as food safety alternatives in the future.

Overall, this study demonstrated the possibility of producing steak‐type cultured meat using 3D printing. DLP‐printing‐based cultured meat production have great potential application in the field of cell cultured meat. Our method may be applicable not only to cultured meat, but also to tissue engineering,^[^ ^16^, ^64^ ^]^ biomedical applications (microfluidic device^[^ ^65^ ^]^ and microvascular construct^[^ ^66^, ^67^ ^]^).

## Conflict of Interest

The authors declare no conflict of interest.

## Supporting information

Supporting Information

Supplemental Video 1

Supplemental Video 2

Supplemental Video 3

Supplemental Video 4

Supplemental Video 5

## Data Availability

Research data are not shared.
