# Supplementary material for: Efficient Myogenic/Adipogenic Transdifferentiation of Bovine Fibroblasts in a 3D Bioprinting System for Steak‐Type Cultured Meat Production
Source: Adv Sci (Weinh). 2022 Oct 3;9(31):2202877. doi: 10.1002/advs.202202877 (PMC9631076; doi:10.1002/advs.202202877)
Supplement: Supplementary file 1 — Supporting Information [file ADVS-9-2202877-s003.pdf]

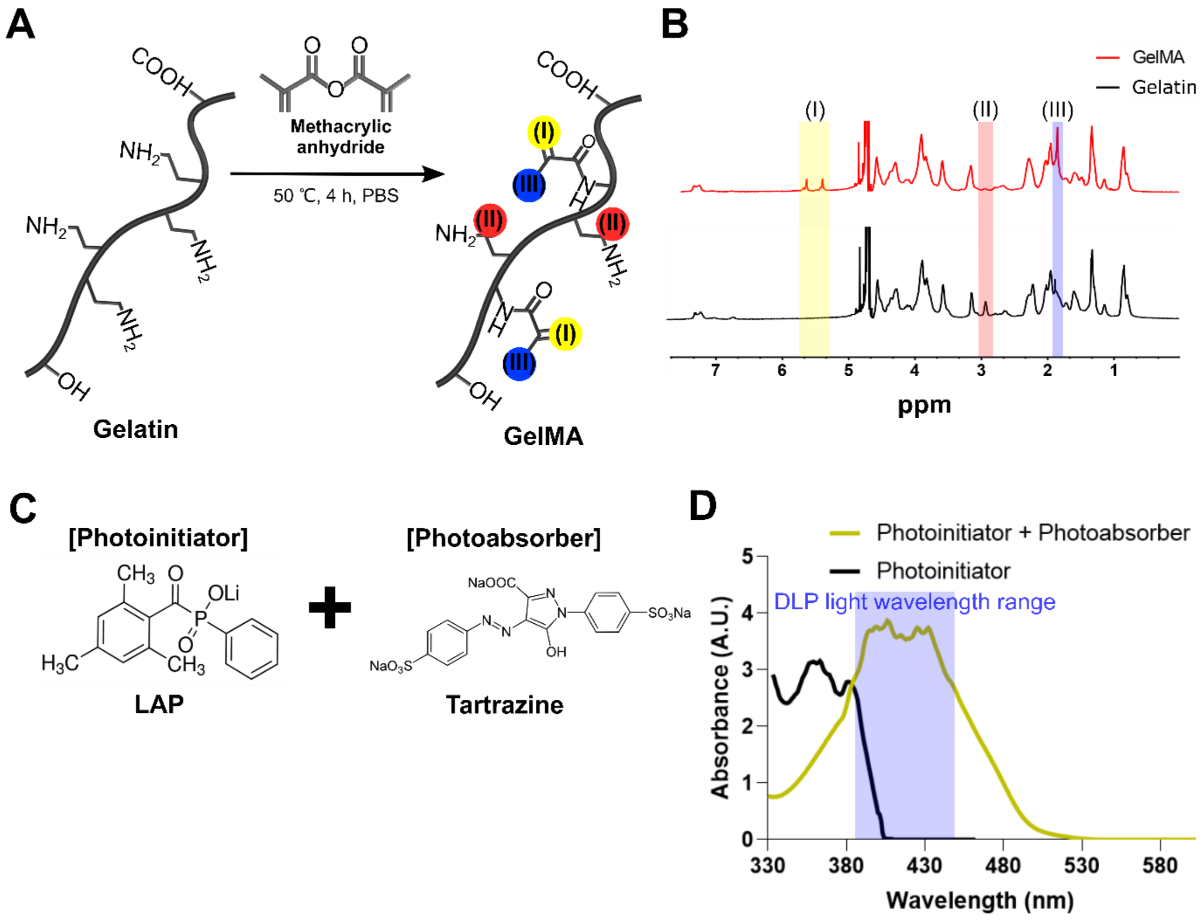

Supporting information 1. (a) GelMA obtained through the reaction of gelatin with methacrylic anhydride. Newly obtained functional groups are shown in yellow (I) and green (III), and the methylene group of lysine in gelatin/GelMA is shown in red (II). (b) ^{1}H NMR of gelatin and GelMA. (c) Chemical structure LAP (photo-initiator) and tartrazine (photoabsorber). (d) Absorbance spectra of photo-initiator and a mixture of photo-initiator and photoabsorber. The shaded area is the light processing based (DLP) light wavelength range.

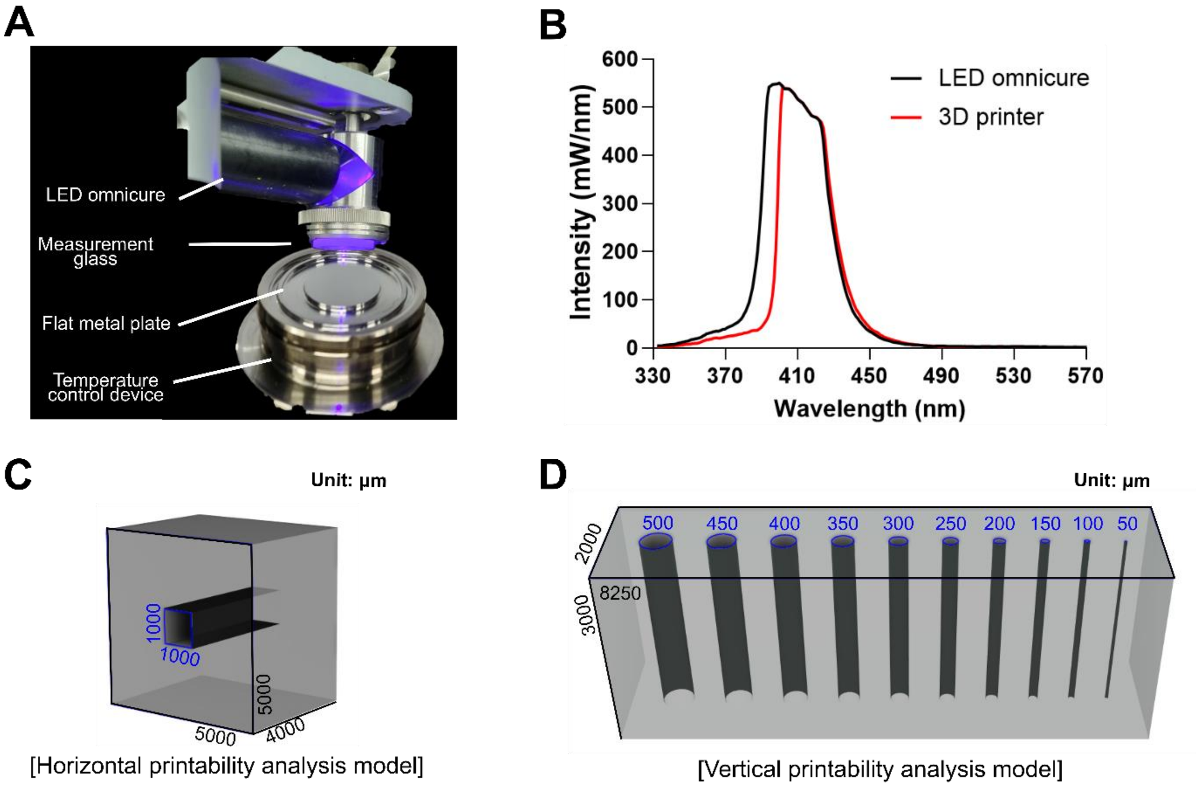

Supporting information 2. (a) Photograph showing the configuration of the photorheometer. (b) Comparison of wavelength bands of DLP 3D printer and LED OmniCure. (c) Horizontal printability analysis model. A square microchannel exists in the center of modeling. (d) Vertical printability analysis model. The modeling contains 10 circulars microchannel of different diameters.

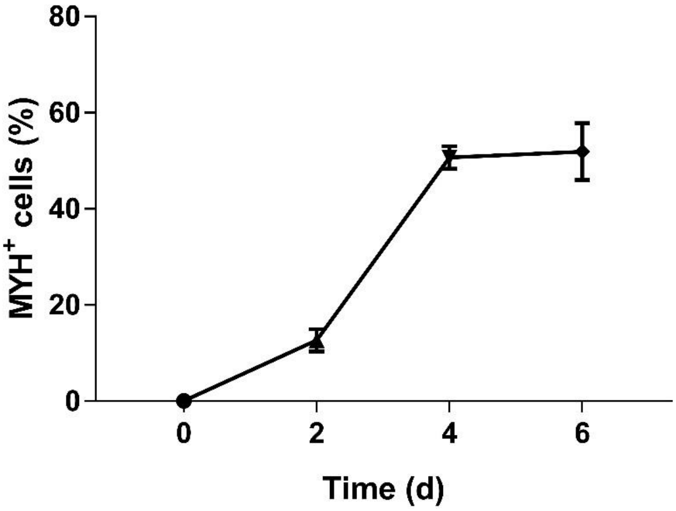

Supporting information 3. (a) MYH-positive cells areas/total areas were quantified using NIH ImageJ software. Data are means ± SDs (n= 4).

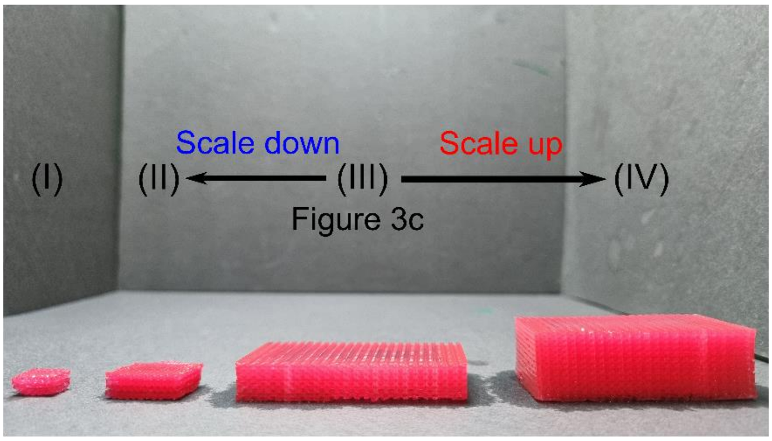

Supporting information 4. Examples of a scale-adjustable steak-type construct. (I) is width, 8.4 mm, length, 10.3 mm, and height, 3.21 mm. (II) is width, 16.8 mm, length, 20.6 mm, and height, 4.82 mm. (III) is width, 34.3 mm, length, 55.3 mm, and height, 9.63 mm. (IV) is width, 34.3 mm, length, 55.3 mm, and height, 19.26 mm.

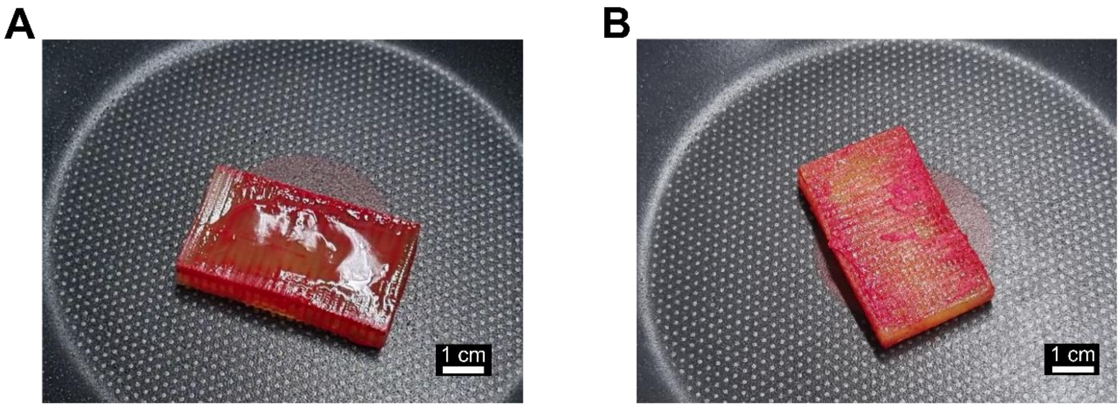

Supporting information 5. Examples of cultured meat cooking process. (a) Raw cultured meat (right before pan-fry). (b) Pan-fried cultured meat.
